# Patterns of *tubb2b* Promoter-Driven Fluorescence in the Forebrain of Larval *Xenopus laevis*

**DOI:** 10.3389/fnana.2022.914281

**Published:** 2022-07-08

**Authors:** Daniela Daume, Thomas Offner, Thomas Hassenklöver, Ivan Manzini

**Affiliations:** Department of Animal Physiology and Molecular Biomedicine, Institute of Animal Physiology, Justus-Liebig-University Gießen, Gießen, Germany

**Keywords:** neuronal β-tubulin, class II β-tubulin, NBT, olfactory bulb, higher olfactory centers

## Abstract

Microtubules are essential components of the cytoskeleton of all eukaryotic cells and consist of α- and β-tubulin heterodimers. Several tissue-specific isotypes of α- and β-tubulins, encoded by distinct genes, have been described in vertebrates. In the African clawed frog (*Xenopus laevis*), class II β-tubulin (*tubb2b*) is expressed exclusively in neurons, and its promoter is used to establish different transgenic frog lines. However, a thorough investigation of the expression pattern of *tubb2b* has not been carried out yet. In this study, we describe the expression of *tubb2b*-dependent Katushka fluorescence in the forebrain of premetamorphic *Xenopus laevis* at cellular resolution. To determine the exact location of Katushka-positive neurons in the forebrain nuclei and to verify the extent of neuronal Katushka expression, we used a transgenic frog line and performed several additional antibody stainings. We found *tubb2b*-dependent fluorescence throughout the *Xenopus* forebrain, but not in all neurons. In the olfactory bulb, *tubb2b*-dependent fluorescence is present in axonal projections from the olfactory epithelium, cells in the mitral cell layer, and fibers of the extrabulbar system, but not in interneurons. We also detected *tubb2b*-dependent fluorescence in parts of the basal ganglia, the amygdaloid complex, the pallium, the optic nerve, the preoptic area, and the hypothalamus. In the diencephalon, *tubb2b*-dependent fluorescence occurred mainly in the prethalamus and thalamus. As in the olfactory system, not all neurons of these forebrain regions exhibited *tubb2b*-dependent fluorescence. Together, our results present a detailed overview of the distribution of *tubb2b*-dependent fluorescence in neurons of the forebrain of larval *Xenopus laevis* and clearly show that *tubb2b*-dependent fluorescence cannot be used as a pan-neuronal marker.

## Introduction

Microtubules, dynamic hollow cylinders composed of α- and β-tubulin heterodimers (Ludueña et al., 1977; Sullivan, 1988; Ludueña and Banerjee, 2008b; Knossow et al., 2020), are essential constituents of the cytoskeleton of all eukaryotic cells (Wade, 2009; Kapitein and Hoogenraad, 2015). They are involved in several fundamental cellular processes, including the maintenance of cell shape, the division of cells during mitosis and meiosis, ciliary and flagellar motion, and various forms of intracellular transport (Ludueña et al., 1977; Nogales, 2000; Muroyama and Lechler, 2017). In developing neurons, microtubules play an essential role in forming the accurate structure of axons, dendrites, and synapses. These functions persist into adulthood to maintain the neurite structure and enable the trafficking of vesicles between organelles (Lasser et al., 2018). A varying number of α- and β-tubulin isotypes have been described in different vertebrate species (Ludueña and Banerjee, 2008a). The analysis of their occurrence in various cell types and tissues has demonstrated a complex pattern of differential distribution of the multiple isotypes (Lewis et al., 1987; Ludueña, 1998; Katsetos et al., 2003). In the brain of many vertebrates, class III β-tubulin is specifically expressed in neurons (Moody, 1989; Lee et al., 1990a,b; Katsetos et al., 1993, 2003; Moody et al., 1996; Latremoliere et al., 2018). However, the African clawed frog (*Xenopus laevis*) steps out of the line as class III β-tubulin could not be detected in the nervous system (Moody et al., 1996). In larval *Xenopus*, another β-tubulin isotype, class II β-tubulin, has been shown to be specially expressed in neurons (Richter et al., 1988; Oschwald et al., 1991). Therefore, the *tubb2b* promoter has been used to establish transgenic frog lines, for example, Xla.Tg(tubb2b:Katushka;cryga:Venus)^EXRC^ (Love et al., 2011) or Xla.Tg(tubb2b:GCaMP6s;Rno.elas:GFP)*^NXR^* (Horb et al., 2019; Offner et al., 2020), where transgenes are specifically expressed in neurons. Although the *tubb2b* promoter is used in many transgenic frog lines, a detailed investigation of its activity pattern at cellular resolution has not been performed yet. In the present study, we analyzed the *tubb2b*-dependent fluorescence expression pattern in the forebrain of larval *Xenopus laevis* on a cellular level. We found *tubb2b*-dependent fluorescence in neurons throughout the *Xenopus* forebrain, that is, the olfactory bulb (OB), parts of the basal ganglia, the pallium, the amygdaloid complex, the optic nerve (OpN), the preoptic area (POA), the prethalamus (PTh), the thalamus (Th), and the hypothalamus (Hyp). Still, clearly, not all neurons in these areas were fluorescently labeled. Together, the results of this work present a detailed overview of the distribution of *tubb2b*-dependent fluorescence in forebrain neurons and show that *tubb2b*-driven fluorescent expression cannot be used as a pan-neuronal marker in the brain of larval *Xenopus laevis*.

## Materials and Methods

### Animals

Transgenic NBT-Katushka γ-cry-Venus [Xla.Tg(tubb2b:Katu shka;cryga:Venus)^EXRC^; Love et al., 2011] and custom-bred NBT-Katushka pax6-GFP [Xla.Tg(tubb2b:Katushka;cryga:Ven us;pax6:GFP;CMV:DsRED)*^Manzini^*] larval *Xenopus laevis* were raised in the animal husbandry facility at the Institute of Animal Physiology of the Justus-Liebig-University of Gießen, Germany. Transgenic NBT-Katushka pax6-GFP frogs were naturally bred from transgenic NBT-Katushka γ-cry-Venus and pax6-GFP CMV-DsRED [Xla.Tg(pax6:GFP;CMV:DsRED)*^Papal^*; Hartley et al., 2001] *Xenopus laevis*. Animals were kept in water tanks of 1.8–7.5 l at a water temperature of 19–22°C and fed with a mix of spirulina and chlorella algae (MS-Tierbedarf). For all experiments, tadpoles of stages 48–52 were used as a representative of the premetamorphic developmental phase (Nieuwkoop and Faber, 1994). All animal procedures were performed following the guidelines of Laboratory Animal Research of the Institutional Care and Use Committee of the Justus-Liebig-University of Gießen (649_M).

### Whole Mount Preparations and Tissue Slicing

All animals were anesthetized in 0.02% MS-222 solution (ethyl 3-aminobenzoate methanesulfonate; TCI Germany), dissolved in tap water, and killed by severing the spinal cord at the level of the brainstem. Tissue blocks containing the whole brain and spinal cord were dissected. Palatial tissue covering the brain was removed, and the blocks were collected in frog Ringer solution (98 mM NaCl, 2 mM KCl, 1 mM CaCl_2_, 2 mM MgCl, 5 mM Na-pyruvate, 5 mM glucose, 10 mM HEPES, pH 7.8, osmolarity of 230 mOsmol/l). For fixation, the samples were immersed in 4% formaldehyde for 1 h and washed with PBS (137 mM NaCl, 2.7 mM KCl, 8 mM Na_2_HPO_4_, 1.4 mM KH_2_PO_4_, dissolved in purified water, pH 7.4). Several blocks were then embedded in 5% of low-melting point agarose (Sigma-Aldrich) and cut into 300-μm-thick coronal slices using a half-automatic vibratome (Leica VT1200S; Leica Biosystems).

### Immunohistochemistry

To help localize neurons with *tubb2b*-dependent fluorescence in the larval forebrain, we performed immunohistochemical stainings against Pax7, calretinin (CR), and tyrosine hydroxylase (TH). All tissue slices were permeabilized using PBST (PBS containing 0.2% Triton-X100; Carl Roth). Unspecific binding sites were blocked with PBST with 2% normal goat serum (NGS; MP Biomedicals) for 1 h at room temperature. The slices were incubated with primary antibodies (1:100) in PBST and 2% NGS at 4°C for 3 days. Katushka was enhanced with anti-tRFP (AB233, polyclonal, derived from rabbit; BioCat) in combination with anti-HuC/D (A-21271, monoclonal, derived from mouse; Thermo Fisher Scientific), anti-calretinin (6B3, monoclonal, derived from mouse; Swant), anti-Pax7 (PAX7, monoclonal, derived from mouse), or anti-tyrosine hydroxylase (22941, monoclonal, derived from mouse; Immunostar). PAX7 was deposited to the DSHB by Kawakami, A. (DSHB Hybridoma Product PAX7). GFP was enhanced with anti-GFP (ab1218, monoclonal, derived from mouse; Abcam). The primary antibodies were washed off with PBS, and the samples were incubated with the secondary antibodies Alexa Fluor 594 goat anti-rabbit (Invitrogen, Thermo Fisher Scientific; 1:100) and Alexa Fluor 488 goat anti-mouse (Invitrogen, Thermo Fisher Scientific; 1:100) in PBS with 2% NGS at 4°C for 3 days. After several washing steps with PBS, the samples were transferred to a recording chamber for multiphoton microscopy (A1R MP; Nikon).

### Image Processing and Data Analysis

All images were recorded at a z-resolution of 1 μm at an excitation wavelength of 780 nm. Image processing was performed using open-source processing software ImageJ (Schindelin et al., 2012).^1^ The raw image data were acquired as multidimensional image stacks. Images obtained from whole mount preparations are shown as maximum projections of all planes of the recorded z-stacks. Images obtained from the coronal slices are shown as individual z-planes. Due to the size of the samples, multiple image stacks were stitched together to show larger brain areas in one picture (Preibisch et al., 2009). Some images were cropped to show details. Brightness and contrast were linearly adjusted in all images. Labeled cells were manually counted using ImageJ ROI Manager in the OB as an example brain region. Mean counts are presented with standard deviation.

## Results

In this study, we characterized the activity pattern of the neuronal β-tubulin (*tubb2b*) promoter in the forebrain of premetamorphic *Xenopus laevis*. We used whole-brain preparations and coronal slices of transgenic tadpoles: NBT-Katushka γ-cry-Venus (*n* = 21 whole brains) and NBT-Katushka pax6-GFP (*n* = 13). Additionally, we performed antibody stainings against Pax7 (*n* = 6), TH (*n* = 8), and CR (*n* = 15) to have landmarks of the main forebrain regions and to describe the precise location of neurons in which the promoter *tubb2b* is active. To gain information about the identity of *tubb2b*-positive cells, CR, TH, and *pax6* were used to differentiate types of interneurons within the OB. Since *pax6* is established as a marker for the main pallial regions of the telencephalon (Stoykova and Gruss, 1994; Puelles et al., 2000; Moreno et al., 2008), we used the NBT-Katushka pax6-GFP transgenic *Xenopus* line to visualize the main parts of the pallium and subpallium. CR is known to be expressed in the central thalamic nucleus and fibers of the lateral forebrain bundle (Morona and González, 2013). We used CR to visualize the boundary between the thalamic nuclei of the diencephalon and the telencephalic hypothalamic areas, which are located ventrally. Within the diencephalon, Pax7 was used to differentiate prosomeres 1–3 (p1–3) as Pax7 expression has been found in p3 and the epiphysis which is part of p2 (Bandín et al., 2013). *tubb2b*-driven fluorescence was found mainly in somata. In some cases, we found additional *tubb2b*-dependent fluorescence in fibers and fiber terminals. We present our results in a rostral–caudal axis, starting from the OB and terminating in the thalamus and hypothalamus. We used the following publications as references to evaluate the expression in specific brain areas: *olfactory bulb*: Nezlin and Schild, 2000; Pinelli et al., 2004; Gaudin and Gascuel, 2005; Weiss et al., 2020; *telencephalon and diencephalon*: González et al., 1994; Marín et al., 1997a,b; Moreno and González, 2003, 2004, 2005; Morona and González, 2013; Bandín et al., 2014.

### Telencephalon

**FIGURE 1:**
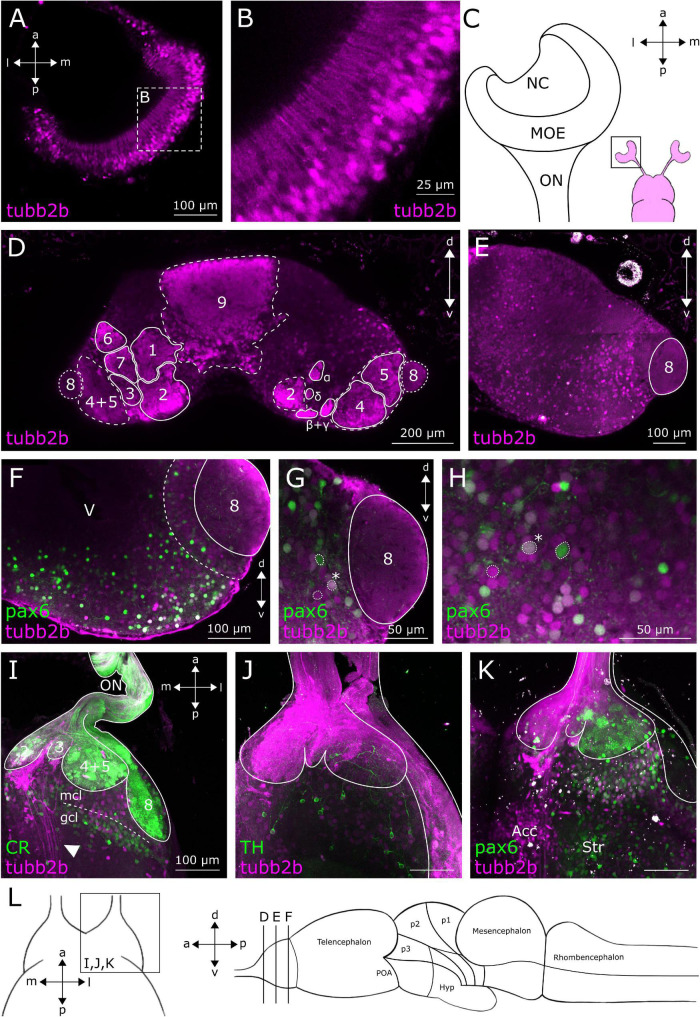
*tubb2b*-dependent fluorescence within the MOE and OB of larval *Xenopus laevis*. **(A)** Dorsal view of a single focal plane of the left main olfactory epithelium (MOE). *tubb2b*-dependent fluorescence (magenta) is present in olfactory receptor neurons. **(B)** Close-up of *tubb2b*-positive olfactory receptor neurons of the MOE. **(C)** Schematic overview of the olfactory organs (magenta). Close-up of the left MOE. **(D)** Coronal slice of the OB (left and right) of a transgenic NBT-Katushka γ-cry-Venus tadpole. *tubb2b*-dependent fluorescence (magenta) is present in axons of ORNs terminating in all known axonal projection fields of the OB (projection fields 1–9 and projection fields α, β, γ, and δ; see Gaudin and Gascuel, 2005). **(E)** Coronal slice of the OB (one side only) of transgenic NBT-Katushka γ-cry-Venus tadpoles and *tubb2b*-dependent fluorescence in cells of the mitral cell layer of the main and accessory (projection field 8) olfactory bulb. **(F–H)** Coronal slices of the OB (one side only) of transgenic NBT-Katushka pax6-GFP tadpoles. **(F)** *tubb2b*- (magenta) and *pax6*-dependent fluorescence (green) in cells in the mitral cell layer. **(G,H)** Close-ups of the mitral cell layer of the main and accessory OB showing *tubb2b*-dependent fluorescence and *pax6*-dependent fluorescence. Examples of cells featuring *tubb2b*-dependent fluorescence only and *pax6*-dependent fluorescence only, and double-fluorescent cells (*) are encircled. **(I–K)** Maximum projection of the whole ventral OB of one brain hemisphere. **(I)** *tubb2b*-dependent fluorescence has not been detected in calretinin (CR; green)-positive cells. *tubb2b*-positive fibers that bypass the OB were found in the intermediate OB (white arrow). **(J)** *tubb2b*-dependent fluorescence has not been detected in tyrosine hydroxylase (TH; green)-positive cells. **(K)** *tubb2b*-dependent fluorescence and *pax6*-dependent fluorescence have been detected in cells of the mitral cell layer. *tubb2b*-dependent fluorescence was also found in the nucleus accumbens (Acc) and *pax6*-dependent fluorescence in the anterior part of the striatum (Str). **(L)** Scheme of the larval brain showing the approximate positions (lines) of the sections shown in **(A–L)**. POA, preoptic area; Hyp, hypothalamus; NC, nasal cavity; ON, olfactory nerve; mcl, mitral cell layer; gcl, granule cell layer; V, ventricle; d, dorsal; v, ventral; a, anterior; p, posterior; m, medial; l, lateral.

**FIGURE 2:**
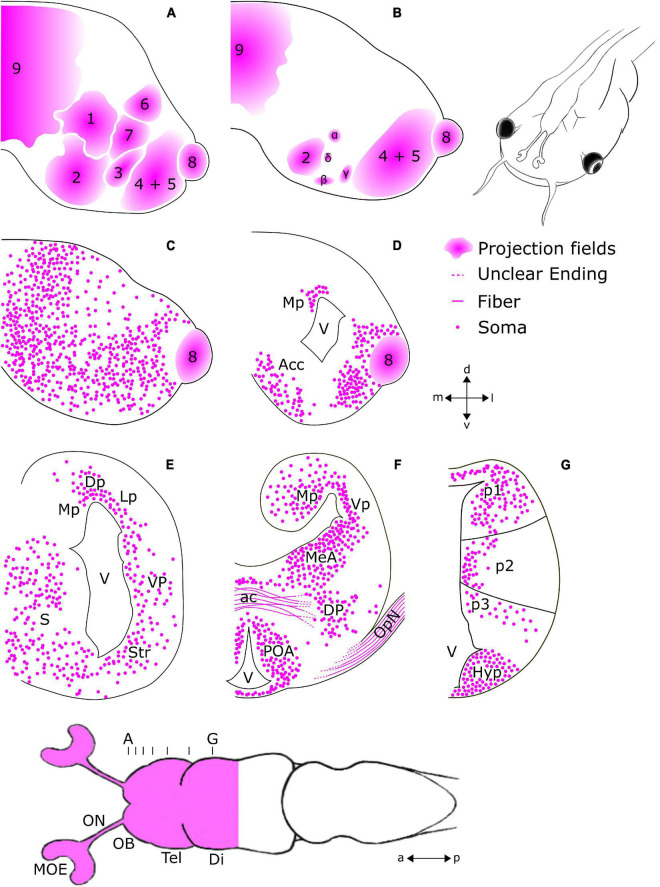
Schematic overview of regions featuring *tubb2b*-dependent fluorescence in the forebrain of premetamorphic *Xenopus laevis*. Ventral view of the larval forebrain and transverse sections of the olfactory bulb (OB; **A–C**), telencephalon (Tel; **A–F**), diencephalon (Di), and hypothalamus **(G)**. The position and density of the cells shown for each region are presented based on observation and not *via* quantitative analysis. **(A,B)** *tubb2b*-dependent fluorescence is present in projection fields of axons arriving from the olfactory epithelium (1–9 + α, β, γ, δ; Gaudin and Gascuel, 2005). **(C,D)** *tubb2b*-dependent fluorescence in cells of the mitral cell layer of the main and accessory olfactory bulbs, the nucleus accumbens (Acc), and pallium. **(E)** *tubb2b*-dependent fluorescence in the medial pallium (Mp), lateral pallium (Lp), dorsal pallium (Dp), ventral pallium (Vp), striatum (Str), and septum (S). **(F)** *tubb2b*-dependent fluorescence in the anterior commissure (ac), medial amygdala (MeA), optic nerve (OpN), dorsal pallidum (DP), and preoptic area (POA). **(G)** *tubb2b*-dependent fluorescence in prosomeres 1–3 (p1–3). Hyp, Hypothalamus; lfb, lateral forebrain bundle; V, ventricle; MOE, main olfactory epithelium; ON, olfactory nerve; a, anterior; p, posterior; d, dorsal; v, ventral; l, lateral; m, medial.

In anurans, the OB is the most anterior telencephalic region (Figure 1L) and is structured in six distinct layers (Byrd and Burd, 1991; Manzini and Schild, 2010; Manzini et al., 2022). The OB, which is connected to the olfactory epithelium *via* the olfactory nerve (ON), is the first relay station of the olfactory system (Manzini and Schild, 2010). We found *tubb2b*-dependent fluorescence in cell bodies of olfactory receptor neurons (ORNs) in the MOE (Figures 1A–C) and associated axons within the ON (Figures 1I–K). Terminals of these axons in the glomerular layer of the main and accessory OB also show *tubb2b*-dependent fluorescence (projection fields 1–9, α, β, γ, and δ; Figures 1D–G, 2A–D). Within the OB of larval *Xenopus laevis*, axon terminals of ORNs form nine large and four small projection fields, which are highly conserved among anurans (Gaudin and Gascuel, 2005; Weiss et al., 2020, 2021). We detected *tubb2b*-dependent fluorescence in axon terminals innervating all of these regions (projection fields 1–9, α, β, γ, and δ; Figures 1D–G, 2A–D) and in a bundle of fibers that project through the OB toward more caudal brain regions (Figure 1I; white arrow).

**FIGURE 3:**
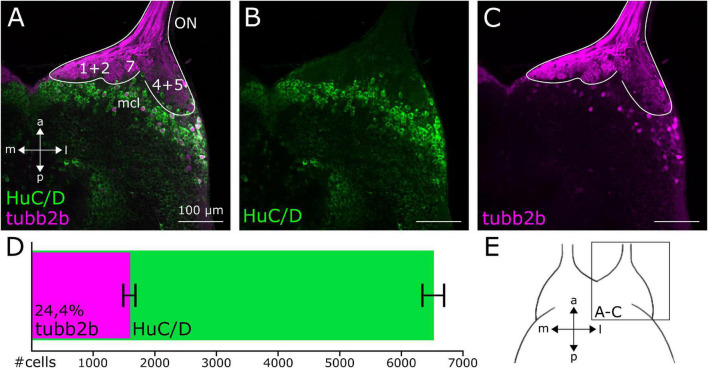
Cell density of *tubb2b*-positive neurons in the OB of premetamorphic *Xenopus* larvae. **(A–C)** Ventral view of a single focal plane of the OB of one brain hemisphere. **(A)** HuC/D-positive cells (green) are present throughout the whole OB. Few HuC/D-positive cells are also *tubb2b*-positive (magenta). **(B)** Pattern of HuC/D-positive cells within the OB. **(C)** *tubb2b*-positive cells are located in the mitral cell layer (mcl) of the OB. **(D)** Number of *tubb2b*-positive cells in comparison to HuC/D-positive cells of the whole OB. Average number of *tubb2b*-positive cells: 1,588 ± 191 (*n* = 10). Average number of HuC/D-positive cells: 6,517 ± 210 (*n* = 3). Cell density of *tubb2b*-positive cells compared to HuC/D-positive cells: 24,4%. **(E)** Scheme of the ventral OB showing the position of the images **(A–C)**. ON, olfactory nerve; a, anterior; p, posterior; m, medial; l, lateral.

**FIGURE 4:**
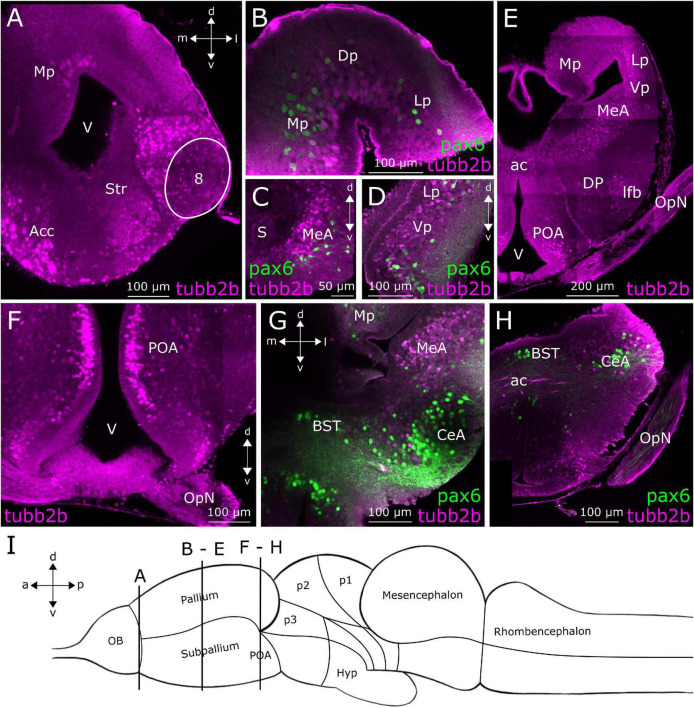
*tubb2b*-dependent fluorescence in the telencephalon of larval *Xenopus laevis*. Coronal sections through the telencephalon of NBT-Katushka γ-cry-Venus **(A,E,F)** and NBT-Katushka pax6-GFP **(B–D,G,H)** transgenic tadpoles. **(A)** *tubb2b*-dependent fluorescence (magenta) in the anterior telencephalon. Stained cells could be detected in the nucleus accumbens (Acc), striatum (Str), accessory OB (projection field 8), and pallium. (**B–D)** Close-ups of various zones of the anterior telencephalon showing *tubb2b*-dependent fluorescence in cells of the dorsal pallium (Dp), ventral pallium (Vp), medial pallium (Mp), lateral pallium (Lp), septum (S), and medial amygdala (MeA). **(E)** Sections through the posterior telencephalon showing *tubb2b*-dependent fluorescence in cells of the Mp, Lp, Vp, MeA, dorsal pallidum (DP), preoptic area (POA), and optic nerve (OpN). **(F–H)** Close-ups of various zones of the ventral posterior telencephalon. **(F)** *tubb2b*-dependent fluorescence in cells of the POA. **(G,H)** No overlap was detected between *tubb2b*- and *pax6*-dependent fluorescence (green) in the POA, ac, and central amygdala (CeA). **(I)** Scheme of the larval brain showing the approximate positions (dashed rectangles) of the sections shown in **(A–H)**. Hyp, hypothalamus; OB, olfactory bulb; BST, bed nucleus of the stria terminalis; V, ventricle; d, dorsal; v, ventral; a, anterior; p, posterior; m, medial; l, lateral.

To visualize all mature neurons of the OB and compare the pattern of *tubb2b*-expressing cells, antibody stainings against HuC/D were done in NBT-Katushka γ-cry-Venus transgenic tadpoles (Figures 3A–C). HuC/D expression was detected in all cell layers of the whole OB (Figures 3A,B; green). We found that *tubb2b* is not expressed in all neurons and surprisingly just in a limited number of cells (Figures 3A,C; magenta). Unlike HuC/D, *tubb2b*-positive cells were found only in the mitral cell layer and close to projection fields 1–9 (Figures 3A,C). For the whole OB, we counted an average number of 6,517 ± 210 HuC/D-positive cells (Figure 3D; green; *n* = 3). In comparison, we calculated an average number of 1,588 ± 191 *tubb2b*-positive cells (Figure 3D; magenta; *n* = 10). As the overall density of *tubb2b*-positive cells compared to HuC/D-positive cells, we calculated an average of 24.4% (Figure 3D). To assign *tubb2b*-dependent fluorescence to the specific bulbar cell types, we performed antibody stainings against CR and TH in transgenic NBT-Katushka γ-cry-Venus tadpoles and used transgenic NBT-Katushka pax6-GFP tadpoles. CR-positive cells were predominantly located in the granule cell layer of the OB (Figure 1I; green) and, to a lesser extent, in the mitral cell layer. All CR-positive cells did not show *tubb2b*-dependent fluorescence (Figure 1I; magenta). TH-positive cells were located in both the mitral cell layer and the periglomerular layer of the OB (Figure 1J; green), but not in the granule cell layer. Again, no cells exhibiting both TH-staining and *tubb2b*-dependent fluorescence were identified (Figure 1J; magenta). *tubb2b*-dependent fluorescence was exclusively located in some, but not all, cells of the mitral cell layer of the main and the accessory OB (Figures 1E–H, 2C,D, 3A,C, 4A; magenta). In this layer, we also found *pax6*-positive cells (Figures 1F–H,K; green), some of them also having *tubb2b*-dependent fluorescence (Figures 1G,H; asterisk). Additionally, a group of cells that show *tubb2b*-dependent fluorescence was located in the nucleus accumbens (Acc; Figures 1K, 2D). This group of cells is also visible in Figure 4A. Laterally, adjacent to Acc, we found *pax6*-positive cells (green) and cells with *tubb2b*-dependent fluorescence (magenta) in the striatum (Str; Figure 1K).

**FIGURE 5:**
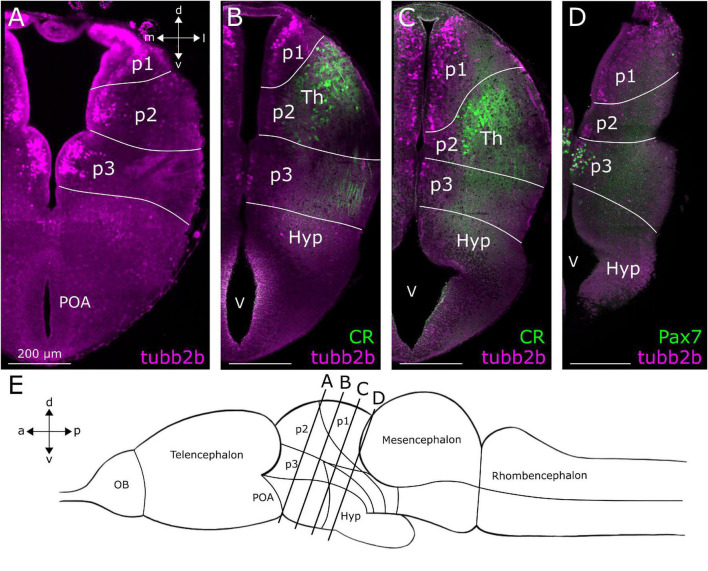
Location of *tubb2b*-dependent fluorescence in the diencephalon of larval *Xenopus laevis*. Coronal sections through the diencephalon of NBT-Katushka γ-cry-Venus transgenic tadpoles. **(A)** *tubb2b*-dependent fluorescence (magenta) in cells of prosomeres 1–3 (p1–3). **(B)** No *tubb2b*-dependent fluorescence could be detected in calretinin-positive cell bodies and fibers (CR; green) of the thalamus (Th). **(C)** *tubb2b*-dependent fluorescence-positive cell bodies in the area of the Th. CR-positive cells and fibers were not *tubb2b*-positive. **(D)** Cells in prosomere 3 (p3) did not show *tubb2b*-dependent fluorescence but could be stained with an antibody against Pax7 (green). **(E)** Approximate positions of the sliced regions are shown in a schematic image of the larval brain (lines). OB, olfactory bulb; Hyp, hypothalamus; POA, preoptic area; V, ventricle; d, dorsal; v, ventral; a, anterior; p, posterior; m, medial; l, lateral.

Figure 4A shows a coronal slice at a posterior position of the anterior telencephalon. At this level in the anterior–posterior axis, we found *tubb2b*-dependent fluorescence (magenta) in cells of the Str, Acc, and accessory OB (projection field 8). First, cells show *tubb2b*-dependent fluorescence in the pallium close to the lateral ventricle (Figures 2D, 4A). A little farther back in the telencephalon, *tubb2b*-dependent fluorescence is located in cells of the medial pallium (Mp; Figures 2E,F, 4B,E), lateral pallium (Lp; Figures 2E, 4B,D,E), and ventral pallium (Vp; Figures 2E,F, 4D,E). *tubb2b*-dependent fluorescence is also present in cells of the medial amygdala (MeA; Figures 2F, 4C,E,G), the central amygdala (CeA; Figures 4G,H), and the septum (S; Figure 4C). Several regions with *tubb2b*-dependent fluorescence also show *pax6*-dependent fluorescence (Figures 4B–D,G,H; green). Thereby, the most prominent group of cells that shows *tubb2b*-dependent fluorescence was present at the level of the CeA (Figures 4G,H). An additional group of cells with *tubb2b*-dependent fluorescence was found close to the dorsal pallidum (DP) and at the position of the lateral forebrain bundle (lfb; Figures 2F, 4E). *tubb2b*-dependent fluorescence-positive fibers were additionally observed in the optic nerve (OpN; Figures 2F, 4E,F,H) and cell bodies in the preoptic area (POA) close to the lateral ventricle (V; Figures 2F, 4E,F). At the level of the anterior commissure (ac), we found *tubb2b*-dependent fluorescence in fibers that project to the contralateral brain hemisphere (Figures 2F, 4H). Moreover, we found that cells of the hypothalamus show strong *tubb2b*-dependent fluorescence (Hyp; Figures 2G, 5B–D).

### Diencephalon

In the diencephalon (Figure 5E), we found *tubb2b*-dependent fluorescence in the subventricular zone of prosomeres 1–3 (p1–3; Figures 2G, 5A–D). In p2, some cells were found in the Th (Figures 5B,C). Similar to the observations made in the telencephalon, *tubb2b*-dependent fluorescence was not found in all neurons of the diencephalon. For instance, at the level of the prethalamus and Th, we found CR-positive fibers that clearly did not show *tubb2b*-dependent fluorescence (Figures 5B,C; green). Additionally, no *tubb2b* expression was seen in CR-positive cell bodies of the Th (Figures 5B,C; green). Moreover, a group of Pax7-positive cells (green) in prosomere 3 (p3) were *tubb2b* negative (Figure 5D).

## Discussion

### *tubb2b*-Dependent Fluorescence Is Not Present in All Neurons of the *Xenopus* Forebrain

β-Tubulin isotypes can occur in different tissues and different subtypes of cells from the same tissue (Eddé et al., 1981; Moura Neto et al., 1983; Sullivan and Cleveland, 1986). In the African clawed frog (*Xenopus laevis*), class II β-tubulin protein is exclusively and widely expressed in the nervous system (Dworkin-Rastl et al., 1986; Oschwald et al., 1991; Bieker and Yazdani-Buicky, 1992) and found to be specially expressed in neurons (Moody et al., 1996). In this study, we monitored the activity of class II β-tubulin *tubb2b* promoter in the forebrain of premetamorphic *Xenopus*. We confirm that it is active in neurons in most main forebrain regions, but we unambiguously found that it is not active in all neurons of these regions. In the OB, *tubb2b*-dependent fluorescence is absent from TH- and CR-immunoreactive interneurons, and only located in the mitral cell layer and periglomerular layer. Cell counts of *tubb2b*-positive cells in comparison to postmitotic HuC/D-positive cells of the OB show that *tubb2b* is only active in 24.4% of all mature neurons in this region. In more posterior areas of the telencephalon, the expression of *tubb2b* is lacking in *pax6*-positive neurons of the CeA, as well as the Mp and Lp. Within the diencephalon, we did not find *tubb2b*-dependent fluorescence in CR-positive neurons and fibers of the Th. Pax7-positive neurons in prosomere 3 also lacked *tubb2b*-dependent fluorescence. The results of the present study show that class II β-tubulin promoter is not active in all forebrain neurons. According to our observations made in the transgenic lines we used, β-tubulin class II cannot be considered a pan-neuronal marker in *Xenopus laevis*.

Polyploidy is a common condition in frogs, and *Xenopus laevis* is one of the few allotetraploid species with four sets of chromosomes (Bisbee et al., 1977; Kobel and Du Pasquier, 1986; Evans et al., 2004; Session et al., 2016). Thus, we cannot fully exclude a mosaic expression of the genome-inserted transgenes in the *Xenopus* lines we investigated. Also, expression patterns of genes and proteins can vary during the development of the animal. In zebrafish (*Danio rerio*), the expression of another tubulin isotype, class I β-tubulin (zβ1), is restricted to the nervous system (Oehlmann et al., 2004). In larvae, zβ1 is expressed in defined peripheral and central nervous system zones that comprise early differentiating neurons. During development, zβ1 decreases, and in adults, its expression is limited to some proliferative brain zones (including the OB) and the olfactory epithelium (Oehlmann et al., 2004). Similarly, *tubb2b*-dependent fluorescence in *Xenopus* larvae is also present in brain regions with proliferative activity, such as the Acc, Lp, Mp, Dp, the ventral POA, and the ventral Hyp (D’Amico et al., 2011). In contrast to the zβ1 expression in zebrafish, in larval *Xenopus*, *tubb2b*-dependent fluorescence was also present in brain regions not described to have proliferative activity, for example, the DP and Th (D’Amico et al., 2011). This characteristic expression pattern suggests that in premetamorphic *Xenopus*, β-tubulin class II could play a role in neurogenesis and have other functions in mature neurons. Because in this study, we focus on premetamorphic *Xenopus* larvae, it must be considered that the *tubb2b* expression in other developmental tadpole stages and in the adult frog could be different. Not only the expression patterns within the same tissue change but also the activity within individual neurons might be different. It has been shown that the expression of β-tubulin isotypes in single mammalian cells can change during development (Guo et al., 2011). In larval *Xenopus laevis*, like in mice, class II β-tubulin is involved in axonal growth and elongation (Moody et al., 1996). In mice, β-tubulins I, II, and III are expressed in neurons of the adult brain (Guo et al., 2011). In cultured neurons of newborn mice, β-tubulin II is no longer expressed in some parts of the cell bodies after differentiation and starts being expressed in neurites, where it might contribute to the regulation of neurite outgrowth (Guo et al., 2011). In the present study, we found that in premetamorphic *Xenopus laevis*, *tubb2b*-dependent fluorescence is mainly located in cell bodies. We observed a very strong *tubb2b*-dependent fluorescence in axons of the ON and OpN. Additionally, we found fibers of the ON that project toward the midbrain and the contralateral brain hemisphere without terminating in the OB. All three structures have their axons cover a relatively long distance, and their cell bodies are located in peripheral organs (Cima and Grant, 1982; Burd, 1991; Pinelli et al., 2004). Because we did not find *tubb2b*-positive neurites of intracerebral neurons, we assume that the expression of *tubb2b* in fibers from the periphery could play a special role and might be important to enable efficient intracellular transport over long distances.

### *tubb2b*-Dependent Fluorescence in Projections From the Olfactory Organs to the Forebrain

In vertebrates, the olfactory system has an exceptional regenerative ability (Graziadei and Dehan, 1973; Graziadei and Monti Graziadei, 1983; Steuer et al., 2014; Yu and Wu, 2017; Calvo-Ochoa et al., 2021). Within the olfactory epithelium, olfactory sensory neurons undergo a constant natural turnover (Graziadei and Monti Graziadei, 1983; Higgs and Burd, 2001; Brann and Firestein, 2014) and have the ability to regrow after a lesion (Schwob, 2002). In premetamorphic *Xenopus laevis*, 7 weeks after ON injury, newborn olfactory sensory neuron axons fully reinnervate the OB (Hawkins et al., 2017). It has been shown that newly formed glomerular clusters and second-order neurons respond to olfactory stimuli during functional calcium imaging (Hawkins et al., 2017). In mammals, class II β-tubulin is associated with axonal growth and elongation (Joshi and Cleveland, 1989). Thus, it might also be involved in the regeneration of ON axons. In *Xenopus* embryos, class II β-tubulin has already been found during the differentiation of pioneering neurons (Moody et al., 1996); moreover, our results show that *tubb2b*-dependent fluorescence is present in the vast majority of axonal projections from the olfactory organs to the OB in premetamorphic *Xenopus*.

Therefore, we observed that some of these axonal projections bypass the OB and terminate in higher brain regions. Most likely, these fibers belong to the EBOS, which has been described in many aquatic vertebrates (Demski and Northcutt, 1983; Schmidt et al., 1988; Von Bartheld and Meyer, 1988; Hofmann and Meyer, 1989; Pinelli et al., 2004). Studies conducted in goldfish (*Carassius auratus*) and salamander (*Triturus alpestris* and *Salamandra salamandra*) show that EBOS fibers target regions like the hypothalamus and are potentially involved in the perception of pheromones (Demski and Northcutt, 1983; Schmidt et al., 1988). In larval *Xenopus laevis*, it has been shown that a particular glomerulus, the so-called γ-glomerulus, is innervated by ipsilateral and contralateral axonal projections originating in the olfactory epithelium (Kludt et al., 2015). These fibers bypass the ipsilateral OB, cross the brain midline *via* the anterior commissure, and project toward the contralateral OB (Kludt et al., 2015). The γ-glomerulus is particular in the sense that it is not innervated by axons of odorant-sensitive receptor neurons, but instead by temperature-sensitive fibers (Kludt et al., 2015). In the present study, we also observed *tubb2b*-dependent fluorescence in fibers projecting through the anterior commissure. Consequently, some of the *tubb2b*-dependent fibers detected in the OB possibly belong to the previously described temperature-sensitive system.

### *tubb2b*-Dependent Fluorescence in Projection Neurons of the Olfactory Bulb

A large number of cells in the mitral cell layer of the OB show *tubb2b*-dependent fluorescence. In the vertebrate OB, the mitral cell layer and the external plexiform layer are populated with cell bodies of projection neurons, the so-called mitral and tufted cells (Nagayama et al., 2014; Kosaka and Kosaka, 2016). The somata of the various interneurons of the OB, for example, juxtaglomerular cells and granule cells, are, respectively, located in the glomerular layer and the granule cell layer (Nagayama et al., 2014; Manzini et al., 2022). In larval *Xenopus laevis*, like in other amphibians (Hoffman, 1963), the mitral cell layer and the external plexiform layer are not clearly disjunct, and thus, an unambiguous assignment of the different bulbar neuron types to the various layers of the OB is not easy (Scalia et al., 1991; Nezlin and Schild, 2000). To overcome this limitation, in the present study, we employed TH and CR immunostainings in the OB of larval NBT-Katushka γ-cry-Venus *Xenopus* to get information about the cell type of cells featuring *tubb2b*-dependent fluorescence. In the OB of larval *Xenopus*, TH antibodies have been shown to stain dopaminergic interneurons in the glomerular and external plexiform layers (González et al., 1994; Boyd and Delaney, 2002), and antibodies against CR have been shown to stain a subset of mitral cells in the mitral cell layer and interneurons (granule cells) in the granule cell layer (Porteros et al., 1997; Castro et al., 2008; Morona and González, 2013). In the present study, we did not find a single TH-positive cell that also shows *tubb2b*-dependent fluorescence. This shows that cells exhibiting *tubb2b*-dependent fluorescence are not dopaminergic TH-immunoreactive interneurons. Similarly, we did not detect a single CR-positive cell featuring *tubb2b*-dependent fluorescence. This rules out that granule cells in larval *Xenopus* express *tubb2b*-dependent fluorescence.

Another study conducted in *Xenopus* identified that Pax6 is expressed in cells of the periglomerular and internal granule cell layers (Bandín et al., 2014). Within the periglomerular layer, Pax6 is present in a subset of dopaminergic interneurons that also express in the TH (Bandín et al., 2014). In this study, we also found that *pax6* promoter-driven fluorescence occurs in cells throughout the glomerular layer, which suggests that these cells might also be dopaminergic neurons. More posteriorly, we found *pax6*-dependent fluorescence in the mitral cell layer of the OB and the accessory OB of larval *Xenopus*, but not in the granule cell layer. In the mitral cell layer, we also found *tubb2b*-dependent fluorescence and a subset of cells featuring both *pax6*- and *tubb2b*-dependent fluorescence. Due to the location of the *tubb2b*-positive cell bodies and the observation that TH- and CR-immunoreactive interneurons are *tubb2b*-negative, we assume *tubb2b* is exclusively expressed in projection neurons. Additionally, the presence of double-positive *pax6* and *tubb2b* cells could be an indication that *pax6* is located in a subset of projection neurons as well. If this is the case, it remains the question of what distinguishes the *tubb2b* from the *pax6* projection neuron population and what role double-positive *tubb2b* and *pax6* cells play in the OB.

### *tubb2b*-Dependent Fluorescence in Higher Olfactory Brain Regions

In *Xenopus laevis*, very little is known about the terminal processing centers of the olfactory system. However, potential target regions of OB neurons, like the Acc and MeA, have been reported in adult *Xenopus laevis* (Marín et al., 1997a,b; Moreno and González, 2003; Moreno et al., 2005). Our study shows that cells expressing *tubb2b* are present in all potential target regions of OB neurons. In the ventral telencephalon, we found *tubb2b*-dependent fluorescence in cell bodies of the Acc, bed nucleus of the stria terminalis (BST), the MeA, and axons of the OpN. The Acc is a main component of the basal ganglia (Marín et al., 1997a,b). Retrograde tracing studies in adult *Xenopus* have shown that the Acc receives input from neurons of the OB, and thus, it might be involved in olfactory information processing (Marín et al., 1997a,b). The BST, which is part of the extended amygdala (Alheid et al., 1995; Moreno et al., 2012), and the MeA are also considered part of the olfactory cortex (Swanson and Petrovich, 1998; Moreno and González, 2003; Moreno et al., 2005). Both might have a similar function as the teleost habenula, which is involved in the control of fear responses (Agetsuma et al., 2010; Lee et al., 2010; Miyasaka et al., 2014). Additionally, we also found *tubb2b*-dependent fluorescence in the CeA, which is the main component of the amygdaloid complex for integration and control of cardiovascular, respiratory, and gastrointestinal functions (Jolkkonen and Pitkänen, 1998; Moreno and González, 2005). Posteriorly in the telencephalon, we have detected *tubb2b*-dependent fluorescence in cells of the pallium, including the LA, which is part of the Vp (Moreno and González, 2004). In teleost fish, the dorsal part of the pallium is homolog to the olfactory cortex of mammals (Wullimann and Mueller, 2004; Miyasaka et al., 2014), and it was shown that in adult *Xenopus laevis*, the pallium is a target of neurons originating from the OB (Moreno et al., 2005). Similar to the MeA, the LA is also a terminal processing center of the olfactory system (Moreno and González, 2004; Moreno et al., 2005). Due to the wiring pattern of the LA, it is discussed to be a homolog of the mammalian basolateral complex (Moreno et al., 2005).

In addition to higher olfactory processing centers, we found that other main forebrain regions also show *tubb2b*-dependent fluorescence. In the telencephalon, *tubb2b*-positive cells are located in the Str, DP, POA, and Hyp. The DP and posterior tuberculum, together with the Str, are considered a homolog of the mammalian nigrostriatal pathway and to regulate different motor functions of the frog (Hoke et al., 2007). The Hyp serves as a control unit for endocrine mechanisms and regulates general functions of the body and behavioral patterns (Bruce, 2009; Domínguez et al., 2013, 2014). In the diencephalon, we observed *tubb2b*-dependent fluorescence in cells of p1–p3. A small number of *tubb2b*-positive cells were visible in the Th of p2. Although the thalamus serves as a relay station for sensory information, it does not process olfactory input (Jones, 2007; Bandín et al., 2015). Although *tubb2b*-dependent fluorescence has been detected in several regions that might be involved in olfactory information processing, we observed that it is clearly not restricted to it. We also found expression in fibers of the OpN, cell bodies in the area of the BST, the S, the POA, and Hyp.

In the past years, the promoter of class II β-tubulin (*tubb2b*) has been used as a tool to create transgenic *Xenopus* lines, like the tubb2b-GCaMP6s Rno.elas-GFP (NXR; Cat# 0.0107; Horb et al., 2019), to drive the expression of the genetically encoded calcium indicator GCaMP6s in the nervous system (Offner et al., 2020). Measurements of neuronal activity have been already possible in the OB and the olfactory amygdala (Offner et al., 2020) and could be performed in all regions (cells) featuring *tubb2b*-dependent fluorescence shown in the present study. Not only the *tubb2b* GCaMP6s transgenic line is a great advantage for functional studies but also our custom-bred NBT-Katushka pax6-GFP line is a great tool to mark and characterize a broad field of brain regions in larval *Xenopus*.

## Data Availability Statement

The raw data supporting the conclusions of this article will be made available by the authors, without undue reservation.

## Ethics Statement

The animal study was reviewed and approved by the Institutional Care and Use Committee of the Justus-Liebig-University of Giessen.

## Author Contributions

DD, TH, and IM conceptualized the study. DD contributed to investigation, formal analysis, visualization, and writing of the original draft. TO provided the NBT-Katushka pax6-GFP transgenic *Xenopus laevis* line. DD, TO, TH, and IM helped with writing – review and editing the manuscript. TH and IM assisted with funding acquisition and resources, and supervision of the work. All authors contributed to the article and approved the submitted version.

## Conflict of Interest

The authors declare that the research was conducted in the absence of any commercial or financial relationships that could be construed as a potential conflict of interest.

## Publisher’s Note

All claims expressed in this article are solely those of the authors and do not necessarily represent those of their affiliated organizations, or those of the publisher, the editors and the reviewers. Any product that may be evaluated in this article, or claim that may be made by its manufacturer, is not guaranteed or endorsed by the publisher.

## Footnotes

1
http://fiji.sc/Fiji

## Abbreviations

1–9, α, β, γ: δ projection fields of axons from the olfactory organ
Acc: nucleus accumbens
ac: anterior commissure
BST: bed nucleus of the stria terminalis
CeA: central amygdala
DP: dorsal pallidum
Dp: dorsal pallium
EBOS: extrabulbar olfactory system
Hyp: hypothalamus
LA: lateral amygdala
lfb: lateral forebrain bundle
Lp: lateral pallium
MeA: medial amygdala
MOE: main olfactory epithelium
Mp: medial pallium
OB: olfactory bulb
ON: olfactory nerve
OpN: optic nerve
p 1–3: prosomeres 1–3
POA: preoptic area
Str: striatum
Th: thalamus
V: ventricle
Vp: ventral pallium.
